# Understanding the Patient Landscape: A Ten-Year Retrospective Examination of Electroconvulsive Therapy in Romania’s Largest Psychiatric Hospital

**DOI:** 10.3390/biomedicines12051028

**Published:** 2024-05-07

**Authors:** Floris Petru Iliuta, Mirela Manea, Aliss Madalina Mares, Corina Ioana Varlam, Radu Mihail Lacau, Andreea Stefanescu, Constantin Alexandru Ciobanu, Adela Magdalena Ciobanu, Mihnea Costin Manea

**Affiliations:** 1 Department of Psychiatry, “Prof. Dr. Alexandru Obregia” Clinical Hospital of Psychiatry, 041914 Bucharest, Romania; florisiliuta@gmail.com (F.P.I.);; 2 Department of Psychiatry and Psychology, Discipline of Psychiatry, Faculty of Dental Medicine, “Carol Davila” University of Medicine and Pharmacy, 010221 Bucharest, Romania; 3 Faculty of Medicine, “Carol Davila” University of Medicine and Pharmacy, 010221 Bucharest, Romania; 4 Neurosciences Department, Discipline of Psychiatry, Faculty of Medicine, “Carol Davila” University of Medicine and Pharmacy, 020021 Bucharest, Romania

**Keywords:** electroconvulsive therapy, socio-demographic data, clinical profile, number of sessions, schizophrenia, recurrent depressive disorder

## Abstract

The aim of this analysis was to investigate the socio-demographic and clinical profile, the effectiveness, and the association of pharmacological treatment in patients who underwent electroconvulsive therapy during the last 10 years in the largest psychiatric hospital in Romania. This study includes 249 patients aged between 18 and 73 years old. Recurrent depression was the most frequent diagnosis for which ECT was performed (T = 96, 38.55%), followed by schizophrenia (T = 72, 28.91%). The most frequent indication for ECT was treatment resistance (T = 154, 61.84%), followed by persistent suicidal ideation (T = 54, 21.68%) and catatonia (T = 42, 16.86%). In 111 (44.60%) cases included in this study, re-hospitalization was required after performing ECT, while 138 (55.40%) participants did not require any further hospital readmissions. Significant differences were found between these groups in terms of socio-demographic data, diagnosis, number of ECT sessions performed, and association of psychotropic medication during and after the procedure, therefore two separate patient profiles were found based on these characteristics. Patients necessitating re-hospitalization post-ECT were mainly males aged 25–44 diagnosed with schizophrenia and underwent a greater number of ECT sessions (7–12), whereas those not requiring re-hospitalization were predominantly females aged 45–64 with recurrent depressive disorder for which 4–6 ECT sessions were performed.

## 1. Introduction

Electroconvulsive therapy (ECT) is a highly effective treatment method that swiftly improves severe psychiatric conditions like depression, mania, schizophrenia, and schizoaffective disorder. It is particularly recommended in cases where patients exhibit catatonia, show resistance to conventional treatments, or are at risk of suicide [1,2]. The main indication for ECT is psychotic depression, where the remission rate can reach 95%, but in non-psychotic melancholic depression, it typically ranges from 55% to 84% [3,4,5,6]. Regardless of its efficacy, ECT is recommended when conventional treatment routes prove ineffective, intolerable, are contraindicated, or the symptomatology shows resistance to medication, especially in cases of acute and severe symptoms where a favorable response to ECT is anticipated [7].

The American Psychiatric Association has established guidelines for ECT use, prioritizing it as the initial treatment in situations necessitating rapid and consistent response, where risks associated with alternative treatments are higher, or when previous drug therapies have been ineffective, and ECT has demonstrated improvement [8].

In terms of contraindications, certain situations of potential risks have been reported. For instance, intracranial lesions or conditions associated with increased intracranial pressure, history of stroke, recent and subsequently complicated myocardial infarction, severe arterial hypertension, presence of risk factors for intracranial hemorrhage, and other conditions associated with a four or five risk score according to the American Society of Anesthesiologists (ASA) classification Top of FormBottom of Form [8].

Concerning potential adverse events, the current method is associated with minimal morbidity and mortality rates. Mortality typically ranges from 2 to 4.5 deaths per 100,000 procedures, which is similar to the anesthetic risk observed in minor surgeries [9]. Significant complications include confusion, delirium, transient headache, muscle soreness, nausea, vomiting, prolonged seizures, dental damage, and circulatory failure [10].

There is significant diversity in ECT practice, varying between countries, within countries, and even within individual healthcare facilities [11]. This considerable variation in ECT practice may stem from uncertainties about its effectiveness and safety [12], challenges in establishing an appropriate performing schedule for the procedure, limited training [13], and also the few peer reviews [14].

In countries such as the United States of America, the United Kingdom, and Australia, where centralized databases are available, calculating the rate of ECT use is straightforward. In Central Eastern European countries, there is significant variation in ECT practice, with Slovakia and the Czech Republic demonstrating high utilization rates, while Hungary and Lithuania exhibit medium rates. Additionally, Western European nations and Scandinavia also show similarly high rates of ECT usage, whereas the rest of the region maintains extremely low utilization rates [15]. However, in numerous countries, including Romania, there is no centralized statistical collection of data regarding ECT use. In these cases, the rate of ECT use can be estimated through questionnaire studies or by compiling individual data from hospitals or institutes [14].

Despite the relevance of ECT for patients with severe mental disorders, there are still few Romanian studies [15,16] investigating patients’ profiles, appropriate indications, outcomes, and undesirable effects.

Taking all of the above into consideration, it is necessary to have a broad perspective on the benefits, risks, and use of ECT in Romania in order to take full advantage of this type of intervention.

## 2. Materials and Methods

### 2.1. Study Design

We conducted a retrospective observational study spanning over a 10-year period, focusing on psychiatric hospitalizations involving 249 patients at the “Prof. Dr. Al. Obregia” Clinical Psychiatric Hospital in Bucharest, Romania. This institute stands as one of the largest psychiatric medical units not only in the country but also in Southeastern Europe, accommodating more than 1200 patients simultaneously. Functioning as a university teaching hospital, it focuses on the diagnosis, treatment, and rehabilitation of patients afflicted with various mental health disorders, originating from Bucharest and the other surrounding counties.

We included only adult patients (aged over 18 years at the time of hospitalization) admitted between January 2013 and December 2023 who underwent ECT for various mental disorders diagnosed according to the Diagnostic and Statistical Manual of Mental Disorders, Fifth Edition (DSM-5) criteria, namely schizophrenia, psychotic disorder, schizoaffective disorder, bipolar affective disorder (BAD), major depressive disorder—single episode, recurrent depressive disorder, and obsessive–compulsive disorder (OCD).

ECT indication was assessed by the attending psychiatrists of the patients. ECT was administered mainly to patients who had responded poorly to pharmacotherapy and to patients who required rapid improvement of life-threatening symptoms, such as persistent suicidal ideation and catatonia. Certain patients, who had previously experienced ECT, requested the treatment voluntarily. Before ECT, all patients, legal representatives, or family members signed informed consent with a prior adequate explanation of the procedure and anesthesia, as well as the anticipated benefits and known risks. The informed consent was obtained accordingly regarding legality and ethical significance for individuals with mental illnesses, strictly following the laws in our country.

All sedatives, hypnotic agents, and anticonvulsants were withdrawn if their main use was mood stabilization and not another associated condition, such as epilepsy, before the administration of ECT. Laboratory testing and required medical evaluations were performed before treatment initiation to identify the relative risks of ECT administration. The approvals of an internal medicine specialist and anesthesiologist were also obtained. All patients received modified ECT; the application was performed with a brief-pulse square-wave ECT device (Thymatron system IV device; Somatics, Inc, Lake Bluff, Ill) in the ECT unit. Standard bifrontotemporal electrode placements were employed for bilateral application. The therapy was conducted on alternate days totaling up to three times per week (Monday, Wednesday, and Friday) with analgo-sedation under the supervision of an anesthesiologist. Standard anesthetic techniques involve the use of a rapid, short-acting induction agent for very brief general anesthesia and a muscle relaxant to prevent serious musculoskeletal complications. Manual ventilation was applied through a facemask during the controlled seizure and the blood oxygen saturation was monitored via pulse oximetry. Respiration was maintained using non-invasive positive-pressure ventilation with 100% oxygen [8].

ECT was stopped either when the patient achieved remission or when the symptoms reached a plateau of improvement at two consecutive treatments. ECT was discontinued if the patient did not respond to the first four to six treatments or developed major complications during ECT, such as severe hypertension, cardiac arrhythmias, and postictal agitation. A thorough clinical and mental state examination was conducted, as per the routine protocol after the ECT procedure, to evaluate the presence of ECT-related side effects. The effectiveness of ECT was determined on the basis of clinical psychiatric evaluation.

The medical records of the “Prof. Dr. Alexandru Obregia” Clinical Psychiatric Hospital stored in an electronic database and from the patients’ clinical charts were examined retrospectively, focusing on several aspects of short-term use of ECT, including age, gender, residence, formal education level, marital and professional status, primary and secondary psychiatric diagnoses, associated sleep disorders, heredo-collateral psychiatric history, duration of disease, side-effects of ECT, ECT indication, number of ECT sessions, antipsychotic and antidepressant medication during and after ECT, and the association of mood stabilizers and benzodiazepines to the main pharmacological treatment after ECT. Records with incomplete data were excluded. All ECT treatment courses were included in order to reflect the utilization of this therapeutic method as best as possible.

The study received approval from the Institutional Research Ethics Committee of the “Prof. Dr. Alexandru Obregia” Clinical Hospital of Psychiatry (approval no. 134/20.12.2023) and was carried out in accordance with the Declaration of Helsinki. Confidentiality and complete anonymity of the identity of the patients were maintained.

### 2.2. Statistical Analysis

The statistical analysis of the data was carried out with the software R version 4.2.2 (R Foundation for Statistical Computing, Vienna, Austria) by applying the chi-squared test of independence in order to study the correlations between the categorical variables. A *p*-value of 0.05 was considered statistically significant. The application of this test illustrates the differences between the frequencies of occurrence of the values of the variables included in the analysis. We decided to apply the chi-square test since all variables are categorical.

## 3. Results

This retrospective review includes 249 patients aged between 18 and 73 years old (M = 44.86, SD = 14.63), 144 (58.53%) female and 105 (41.46%) male. Most of the participants (T = 108, 43.37%) were in the age group of 45–64 years. Medical records showed that 216 (86.74%) patients lived in urban areas and 33 (13.25%) in rural areas. Regarding marital status, 129 (51.80%) of the participants were single and 114 (45.8%) of the participants were married, with only 6 (2.41%) living in concubinage. Most of the participants, 129 (51.80%) in total, were in need of specialized social assistance, 66 (26.5%) were employed, 99 (39.75%) attended high school, and 51 (20.48%) attended university. A number of the participants, 225 (90.36%), lived with their families (Table 1).

Ninety-six (38.55%) patients who performed ECT were diagnosed with recurrent depression disorder, 72 (28.91%) were diagnosed with schizophrenia, 45 (18.07%) had a diagnosis of bipolar affective disorder—depressive episode (at the time of hospitalization), 15 (6.02%) had a diagnosis of schizoaffective disorder, 9 (3.61%) of the participants had a diagnosis of psychotic disorder, 6 (2.40%) had a diagnosis of OCD, and 6 (2.40%) had bipolar affective disorder and a single depressive episode. Also, 150 patients (60.24%) had a secondary psychiatric diagnosis, and 147 (59.03%) participants had associated sleep disorders (Table 2).

In addition, 135 (54.21%) participants had the duration of the disease between 5 and 15 years, this being the median of the disease-duration variable. Fifty-seven (22.89%) participants had a disease duration of less than 5 years and 57 (22.89%) had a disease duration of more than 5 years. Regarding the indications for performing ECT, 153 (61.44%) participants presented pharmacological treatment resistance, 54 (21.68%) had persistent suicidal ideation, and 42 (16.86%) had catatonia (Table 2).

The median number of ECT sessions was six in our sample. Most patients (105, 42.16%) underwent between 4 and 6 sessions, and 99 (39.75%) underwent between 7 and 12 sessions. The side effects of ECT, namely hypertension, temporal amnesia, and confusion, which did not lead to treatment cessation, occurred in 78 (31.32%) participants (Table 2). There were no ECT-related fatal events during the survey. Regarding the effectiveness of ECT, 111 (44.60%) participants were re-hospitalized after the ECT sessions, while 138 (55.40%) did not need re-hospitalization.

Regarding medication, 228 (91.56%) participants received antipsychotics during the ECT sessions, while 162 (65.06%) received antidepressants. A total of 186 participants (74.69%) with antipsychotic medication also received the same drug after the ECT sessions, and most of them, 84 (33.73%), were prescribed the same dose as before ECT. Among the participants who received an antidepressant during the ECT sessions, 159 participants (63.85%) were given the same antidepressant before ECT, and for 96 (38.55%) of them, the same dose was maintained after the course of ECT treatment (Table 3).

Therefore, most participants who required re-hospitalization after undergoing ECT had the following characteristics: they were between 25 and 44 years old (T = 51, 58.62%), they were predominantly males (T = 57, 51.35 %), came from urban areas (T = 87, 78.37%), and most of them graduated high school (T = 39, 35.135%), were single (T = 57, 51.35%), and lived with their family (T = 99, 89.18%). Also, 51 participants (45.94%) who were re-hospitalized after ECT were under social assistance provided by the government, with a primary diagnosis of schizophrenia (T = 42, 37.83%), presented sleep disorders (T = 72, 64.86%), and had a secondary psychiatric diagnosis (T = 60, 54.05%) without psychiatric heredo-collateral history (81, 72.93%). The majority of them had a duration of the disease of between 5 and 15 years (T = 60, 54.05%), had no side effects during ECT (T = 75, 67.56%), had treatment resistance as the primary indication for ECT (T = 78, 70.27%), and underwent between 7 and 12 sessions of ECT (T = 54, 48.64%). Most of the patients who required rehospitalization after ECT received antipsychotic (T = 96, 86.48%) or antidepressant (T = 63, 56.75%) treatment during ECT, and the majority remained on the same medication after ECT. A relatively small proportion (T = 18, 16.21%) needed the association of a mood stabilizer to the main pharmacological treatment, but almost half of them (T = 45, 40.54%) needed the association of a benzodiazepine after ECT.

In addition, most patients who did not need to be re-admitted to our hospital after performing ECT had the following characteristics. The majority of them (T = 72,52.17%) were between 45 and 64 years old, female (T = 90,65.21%), came from the urban environment (T = 129, 93.47%), graduated high school (T = 60,43.47%), lived with their family (T = 126, 91.30%), and needed social aid (T = 78,56.52%). Regarding the diagnosis, most of them were diagnosed with recurrent depression (T = 63, 45.65%), had another secondary psychiatric diagnosis (T = 90, 65.21 %), mostly sleep disorders (T = 75, 54.34%), and had a lack of heredo-collateral psychiatric history (T = 90, 65.21%).

The majority of patients who did not require re-admission after ECT had a duration of the disease between 5 and 15 years (T = 75, 54.34%), and underwent between 4 and 6 sessions (T = 72, 52.17%) with no side effects (T = 96, 69.56%). The main indication for ECT is resistance to treatment (T = 75, 54.34%). Furthermore, most of them received an antipsychotic (T = 132, 95.65%) and an antidepressant during ECT (T = 99, 71.73%), at the same dose (for antipsychotic T = 51, 35.95%; for antidepressant T = 69, 50.00%), with an association of benzodiazepines (T = 84, 60.87%) after ECT treatment (Table 1 and Table 2).

The chi-squared test illustrated the fact that there are significant differences between the patients who no longer needed hospitalization after the ECT sessions and those who were re-hospitalized in terms of age, gender, background, level of education, marital and professional status, type of diagnosis, ECT indicators, treatment compliance, number of sessions, and doses of antipsychotic and antidepressant (In the case of the antipsychotic, the same dose. In the case of the antidepressant, the higher dose), *p* < 0.05 (Table 4).

## 4. Discussion

The majority of the patients performing ECT were between 45 and 64 years old in our study sample, an aspect that is consistent with the literature [17,18]. The majority of the patients received about 6–10 sessions of ECT, as shown in other studies [19,20]. 

The number of ECT sessions given to patients worldwide varies widely, from 1 to 22, which could be due to variations in patient demographics, clinical profiles, or differences in available resources and practices [21]. The disproportionate utilization of ECT between urban and rural settings, with 87% of patients hailing from urban areas compared to 13% from rural regions, may be attributed to enhanced access to specialized mental health facilities, greater availability of trained professionals, and heightened awareness and acceptance of ECT within urban populations. Economic factors, cultural attitudes, and logistical challenges in rural areas may further contribute to this disparity in ECT utilization rates. In addition, the prior literature findings have highlighted inequities in access to ECT treatment, as reflected by nonclinical factors such as being unmarried, lower educational attainment, and lack of proximity to ECT treatment facilities, which are associated with a lower likelihood of receiving treatment [22,23,24].

In our retrospective study, more women received ECT than men, which is similar to the Western literature [25,26,27,28], but different from several Asian and African countries [25,29,30,31]. This difference may be caused by the fact that a larger number of women came to our hospital for psychiatric treatment or by the possibility of gender bias in indicating ECT. No significant gender differences were observed in the number of ECT sessions required to induce an adequate response across various psychiatric diagnoses. ECT-induced clinical response remains independent of gender, reaffirming its efficacy in both men and women, despite variations in indication and diagnosis [32,33]. Currently, both the existing literature and our findings support the notion that, while certain gender differences persist in the indication and diagnosis of electroconvulsive therapy, there appears to be no discrepancy in response to the treatment itself [32]. However, there remains a pressing need for prospective studies to delve into the underlying reasons behind these observed differences.

Our study found that marital status (being single) may be associated with a higher likelihood of receiving ECT compared to married or partnered individuals. Possible explanations for this association, among other factors, include the severity of the disease and the urgent need for treatment in case of persistent suicidal ideation and catatonia. It is noteworthy that this aspect derived from our study contradicts most previous research, where married patients were more likely to undergo ECT [34,35,36,37]. A plausible explanation for this discrepancy could be the evolving social attitude towards mental illness and ECT treatment, which has seen increased acceptance in recent years. We found that patients living with their families were more likely to receive ECT than those living alone. Possible reasons for this finding include greater access to healthcare, stronger social support, and reduced stigma. Family support can come in various forms, such as emotional encouragement, practical assistance, and help with managing treatment side effects, which could encourage patients to consider ECT as a treatment option.

Individuals who needed social aid constituted the largest occupational group among patients receiving ECT in our study. These patients often suffered from chronic conditions and benefited from health insurance that covered hospitalization and free therapeutic procedures. This result is consistent with the studies in the literature targeting patients in the psychotic range [38], but contrary to information revealed by studies on depression, in which most patients are employed and have a higher education [22].

Among our patients, recurrent depression disorder (T = 96, 38.55%) and schizophrenia (T = 72, 28.91%) were the most common diagnostic indications for performing ECT. This finding is similar to the studies reported from Western countries, where affective disorders are the most common indication for ECT [25,26,27,28], and some studies from Asian countries suggest that schizophrenia and psychotic disorders are the most common indications for ECT [18,25,30,39].

The most common clinical indication of ECT in our sample was resistance to pharmacological treatment (T = 153, 61.44%), similar to the previous studies from Europe [25,34,40], followed by persistent suicidal ideation (T = 54, 21.68%). Augmentation of medication with ECT was considered when the clinical status required a rapid response to treatment and to increase the effectiveness of medication. Treatment resistance or no response to pharmacological therapy was considered when there was minimal response to medications after 2 weeks of adequate administration. Suicide is a leading cause of death among psychiatric patients, and the rapid relief of severe depression, mania, and psychosis by ECT is accompanied by a rapid reduction in suicidality. The third main indication in our patients was catatonia (T = 42, 16.87%), which may often result in difficulties regarding proper nutrition and the use of oral medication. Catatonic symptoms and suicidal ideation show an early and good response to ECT [41]. Hence, ECT has an important role when an urgent therapeutic response is needed when facing significant vital risk in the presence of suicidality and catatonic symptoms [42]. With regard to adverse effects, we identified that a small number of patients experienced hypertension, temporary amnesia, and moderate agitation after ECT. According to a recent epidemiological study of Medicare data, patients who underwent ECT had lower all-cause mortality for up to 1 year from post-hospital discharge compared to the control group. Notably, there was a significant reduction in suicide rates following ECT, although no difference was observed at the 1-year follow-up [43].

Regarding the effectiveness of ECT, our study revealed that, out of the participants, 44.60% required re-hospitalization following the ECT sessions. In a recent medical study examining severe depression patients, it was found that the rehospitalization rates were notably high, with 43% occurring within 6 months and 58% within 2 years following the initiation of ECT [44]. Conversely, in another study focusing on schizophrenia patients, those who underwent ECT exhibited markedly lower readmission rates compared to their counterparts who did not receive ECT. Specifically, within the ECT group, the 3- and 6-month readmission rates stood at 11.37% and 17.94%, respectively, significantly lower than the rates observed in patients who did not undergo ECT (18.79% and 29.36%, respectively) [45].

In our study, there are significant differences between the patients who no longer needed hospitalization after the ECT procedure and those who were hospitalized again after performing ECT in terms of socio-demographic data, diagnosis, number of performed ECT sessions, and the association of psychotropic drugs during and after the procedure (Table 5).

Therefore, the patients who needed hospital readmission after undergoing ECT had the following characteristics; most of them were aged 25–44 years, male, and had a predominant diagnosis of schizophrenia. Regarding medication, patients who received antipsychotics (AP) experienced lower rates of readmission afterward. Furthermore, those who continued with the same dose or received a higher dose of antipsychotics post-ECT had lower rates of readmission. In patients requiring rehospitalization, it was identified that a higher number of ECT sessions were required (7–12), as opposed to those with depressive disorder, where 4–6 sessions were performed.

On the contrary, patients who did not need further hospitalization after ECT and who were compliant with the recommended psychotropic treatment after undergoing ECT were in the age category of 45–64 years, predominantly female, and had a diagnosis of recurrent depressive disorder. ECT has repeatedly been demonstrated to be safe for elderly patients [46,47]. A recent study demonstrated that ECT led to a swift reduction in depressive symptoms within the initial 10 treatments, persisting for nearly two years during maintenance treatment [48]. In our study, after undergoing 4–6 sessions of ECT, the patients experienced significant improvement in symptoms, particularly in reducing persistent suicidal ideation. Once again, ECT proved to be more effective than pharmacotherapy in treating depression, sustaining the data from the literature [49,50]. Similarly, patients who received antidepressants (AD) had lower rates of readmission afterward. Continuing with the same dose of antidepressants post-ECT was associated with decreased readmission rates. However, at a higher dose, readmission increased, while at a lower dose, readmission remained the same. Patients who received benzodiazepines after ECT presented a significant decrease in this rate. These findings are corroborated by a study by Al Saadi et al. that investigated a subset of psychiatric patients undergoing ECT to check if there were differences in demographic and clinical outcomes between subgroups [35]. Cluster one comprised older women with depressive disorders who received fewer ECT sessions. In contrast to cluster one, patients in cluster two were predominantly young men, most of whom had schizophrenia and required more sessions of ECT [35], results that are consistent with the findings of our study.

This study has several limitations that should be acknowledged. First of all, due to the retrospective nature of the research, we could not directly assess the patients in order to establish the diagnosis using a structured clinical interview, and no rating scale could be used to monitor the effectiveness of ECT in terms of treatment response and clinical improvement. Secondly, there are some variables that do not have a relatively equal number within the formed categories, such as the duration of the disease and the diagnosis. At the same time, we used the median of the summary of sessions and the median of the duration of the disease because we intended to identify the common characteristic of all patients enrolled in the present study. When referring to patients with psychiatric disorders, one might be facing extreme values, and, to avoid managing these widely ranging values, we used the median of the group. Thus, our results can be specific to the categories taken into account, with their degree of generality not being very high. Third, the diagnoses were established by different psychiatrists. It is important to mention that the psychiatrists from our hospital are well-familiarized with the DSM-5 criteria and encoding system. Moreover, we analyzed the diagnosis at discharge, which, unlike an emergency/admission diagnosis, is assigned after multiple and thorough evaluations. Also, the studied population comprised a small sample size, with the lack of a comparison group. Finally, although this research was conducted at the largest mental health hospital in Romania, it represents only a single psychiatric center, limiting the generalizability of our findings to the broader Romanian mental healthcare system. Studies designed prospectively, with larger sample sizes comparing the data of different institutions in the country (university hospitals, state hospitals, and research and teaching hospitals) would give more information with regards to the practice of ECT in psychiatric patients in Romania. There is a need for longitudinal studies to assess clinical improvement using rating scales before and after ECT. Comparative case–control studies are needed to compare the clinical improvement in patients who received ECT versus patients who did not receive ECT and other adult populations.

## 5. Conclusions

In conclusion, our study underscores the considerable diversity in demographic and clinical profiles among psychiatric patients undergoing electroconvulsive therapy (ECT). This diversity highlights the imperative for personalized approaches to ECT treatment, tailored to individual patient characteristics, to optimize both efficacy and safety in managing severe mental disorders.

The heterogeneity observed in our patient cohort elucidates why previous investigations into ECT outcomes among psychiatric populations have yielded inconsistent results. Factors such as age, gender, psychiatric diagnosis, comorbidities, and treatment history contribute to this variability, necessitating a nuanced understanding of patient-specific considerations.

Our findings provide valuable guidance for clinicians tasked with refining ECT protocols. By recognizing and accounting for the multifaceted nature of patient demographics and clinical presentations, clinicians can develop tailored ECT strategies that address the unique needs and circumstances of each patient subgroup. This personalized approach holds the potential to enhance treatment outcomes and minimize adverse events, thereby optimizing the therapeutic benefits of ECT for individuals with severe mental illness.

## Figures and Tables

**Table 1:** Socio-demographic data of the participants.

Variables	Values	Rehospitalized after Performing ECT					
		Yes		No		Total Sample	
		N	%	N	%	N	%
Age category	18–24 years	15	13.51%	15	10.87%	30	12.04%
	25–44 years	51	45.94%	36	26.08%	87	34.94%
	45–64 years	36	32.43%	72	52.17%	108	43.37%
	>65 years	9	8.10%	15	10.87%	24	9.63%
Gender	Male	57	51.35%	48	34.78%	105	41.46%
	Women	54	48.64%	90	65.21%	144	58.53%
Residence	Urban	87	78.37%	129	93.47%	216	86.74%
	Rural	24	21.62%	9	6.52%	33	13.25%
Years of study	6	0	0.00%	3	2.17%	3	1.20%
	8	3	2.70%	3	2.17%	6	2.41%
	10	15	13.51%	9	6.52%	24	9.63%
	11	6	5.40%	3	2.17%	9	3.61%
	12	39	35.13%	60	43.47%	99	39.75%
	13	6	5.40%	15	10.87%	21	8.43%
	14	3	2.70%	0	0.00%	3	1.20%
	15	21	18.91%	30	21.73%	51	20.48%
	16	15	13.51%	12	8.69%	27	10.84%
	18	3	2.70%	3	2.17%	6	2.41%
Marital status	Married	48	43.24%	66	47.82%	114	45.73%
	Single	57	51.35%	72	52.17%	129	51.80%
	Concubinage	6	5.40%	0	0.00%	6	2.41%
Housing status	Single	12	10.81%	12	8.69%	24	9.63%
	With family	99	89.18%	126	91.30%	225	90.36%
Professional status	Unemployed	9	8.10%	21	15.21%	30	12.04 %
	Retired	3	2.70%	3	2.17%	6	2.41%
	Social aid	51	45.94%	78	56.52%	129	51.80%
	Student	12	10.81%	6	4.34%	18	7.22%
	Employee	36	32.43%	30	21.73%	66	26.50%
Total patients		111	44.57%	138	55.42%	249	

N = number of patients; % = percent of patients.

**Table 2:** Characteristics of diagnosis and ECT procedures in participants.

Variables	Values	Rehospitalized after Performing ECT					
		Yes		No		Total Sample	
		N	%	N	%	N	%
Diagnosis	Schizophrenia	42	37.83%	30	21.73%	72	28.91%
	Psychotic disorder	6	5.40%	3	2.17%	9	3.61%
	Schizo-affective disorders	9	8.10%	6	4.34%	15	6.02%
	BAD—depressive episode	18	16.21%	27	19.56%	45	18.07%
	BAD	0	0.00%	3	2.17%	3	1.20%
	Single episode depression	0	0.00%	3	2.17%	3	1.20%
	Recurrent depression	33	29.73%	63	45.65%	96	38.55%
	OCD	3	2.70%	3	2.12%	6	2.40%
Secondary psychiatric diagnosis	no	51	45.94%	48	34.78%	99	39.75%
	yes	60	54.05%	90	65.21%	150	60.24%
Sleep disorder	no	39	35.13%	63	45.65%	102	40.96%
	yes	72	64.86%	75	54.34%	147	59.03%
Heredo-collateral psychiatric history	no	81	72.97%	90	65.21%	171	68.67%
	yes	30	27.02%	48	34.78%	78	31.32%
Duration of disease	<5 years	30	27.02%	27	19.56%	57	22.89%
	5–15 years	60	54.05%	75	54.34%	135	54.21%
	>15 years	21	18.91%	36	26.08%	57	22.89%
Side effects of ECT ECT indication	no	75	67.56%	96	69.56%	171	68.67%
	yes	36	32.43%	42	30.43%	78	31.32%
	resistence to treatament	78	70.27%	75	54.34%	153	61.44%
	suicide ideation	15	13.51%	39	28.26%	54	21.68%
	catatonia	18	16.21%	24	17.39%	42	16.86%
Number of ECT sessions	1–3 sessions	18	16.21%	18	13.04%	36	14.45%
	4–6 sesions	33	29.73%	72	52.17%	105	42.16%
	7–12 sesions	54	48.64%	45	32.60%	99	39.75%
	>13sesions	6	5.40%	3	2.17%	9	3.61%
Total		111	44.57%	138	55.42%	249	

N = number of patients; % = percent of patients.

**Table 3:** Association of medication during and after ECT.

Variables	Values	Rehospitalized after Performing ECT					
		Yes		No		Total Sample	
		N	%	N	%	N	%
Use of antipsychotic medication during ECT	no	15	13.51%	6	4.34%	21	8.43%
	yes	96	86.48%	132	95.65%	228	91.56%
Use of antidepressant medication during ECT	no	48	43.24%	39	28.26%	87	34.94%
	yes	63	56.75%	99	71.73%	162	65.06%
Use of the same antipsychotic after ECT	no	36	32.43%	27	19.56%	63	25.30%
	yes	75	67.56%	111	80.43%	186	74.69%
Dosage of the same antipsychotic after ECT	equal dose	33	29.73%	51	36.95%	84	33.73%
	lower dose	24	21.62%	24	17.39%	48	19.27%
	higher dose	30	27.02%	33	23.91%	63	25.30%
	not necessary	24	21.62%	30	21.73%	54	21.68%
Use of the same antidepressant after ECT	no	51	45.94%	39	28.26%	90	36.14%
	yes	60	54.05%	99	71.73%	159	63.85%
Dosage of the same antidepressant after ECT	equal dose	27	24.32%	69	50.00%	96	38.55%
	lower dose	12	10.81%	12	8.69%	24	9.63%
	higher dose	21	18.91%	15	10.87%	36	14.45%
	not necessary	51	45.94%	42	30.43%	93	37.34%
Association of mood stabilizer after ECT	no	93	83.78%	96	69.56%	189	75.90%
	yes	18	16.21%	42	30.43%	60	24.09%
Association of benzodiazepines after ECT	no	66	59.45	54	39.13%	120	48.19%
	yes	45	40.54%	84	60.87%	129	51.80%
Total		111	44.57%	138	55.42%	249	

N = number of patients; % = percent of patients.

**Table 4:** Chi-squared tests.

	Value	df	*p*
Χ^2^ age	13.315	3	0.004
Χ^2^ gender	6.925	1	0.008
Χ^2^ residence	12.201	1	<0.001
Χ^2^ study	15.994	9	0.047
Χ^2^ matital status	7.750	2	0.021
Χ^2^ housing status	0.316	1	0.574
Χ^2^ professional status	12.817	5	0.025
Χ^2^ diagnostic	18.060	7	0.012
Χ^2^ secondary psych dg	3.201	1	0.074
Χ^2^ sleep disorder	2.814	1	0.093
Χ^2^ heredocollateral psych ant	1.720	1	0.190
Χ^2^ duration	2.878	2	0.237
Χ^2^ side effects	0.114	1	0.736
Χ^2^ ECT indicators	8.758	2	0.013
Χ^2^ sesions	13.535	3	0.004
Χ^2^ compliance	225.802	2	<0.001
Χ^2^ AP equal dose	18.597	3	<0.001
Χ^2^ AP lower dose	4.429	3	0.219
Χ^2^ AP higher dose	14.162	3	0.003
Χ^2^ AP it’s not necessary	4.590	3	0.204

**Table 5:** Profile of patients who did and did not require readmission after ECT.

Characteristic	Patients Requiring Readmission After ECT	Patients Not Requiring Readmission After ECT
Age Category	25–44 years	45–64 years
Gender	Male	Female
Predominant Diagnosis	Schizophrenia	Recurrent depressive disorder
ECT Sessions Required	7–12	4–6
Post-ECT Medication	Antipsychotics (AP)	Antidepressants (AD), Benzodiazepines
Effect on Readmission Rates	Lower rates of readmission	Decreased readmission rates, significant decrease in association with benzodiazepines
Continuing Dosage of Medication	Same or higher dosage of antipsychotics post-ECT	Same dosage of antidepressants post-ECT, significant decrease with benzodiazepines
Relationship to Suicidal Ideation	Not specified	Significant improvement, particularly in reducing persistent suicidal ideation

## Data Availability

All information included in this article is documented by relevant references.
